# Having Difficulties Reading the Facial Expression of Older Individuals? Blame It on the Facial Muscles, Not the Wrinkles

**DOI:** 10.3389/fpsyg.2021.620768

**Published:** 2021-06-04

**Authors:** Sabrina N. Grondhuis, Angela Jimmy, Carolina Teague, Nicolas M. Brunet

**Affiliations:** Department of Psychology and Neuroscience, Millsaps College, Jackson, MS, United States

**Keywords:** facial expressions, facial muscles, aging related, artificial aging, emotional expressions, emotion recognition

## Abstract

Previous studies have found it is more difficult identifying an emotional expression displayed by an older than a younger face. It is unknown whether this is caused by age-related changes such as wrinkles and folds interfering with perception, or by the aging of facial muscles, potentially reducing the ability of older individuals to display an interpretable expression. To discriminate between these two possibilities, participants attempted to identify facial expressions under different conditions. To control for the variables (wrinkles/folds vs facial muscles), we used Generative Adversarial Networks to make faces look older or younger. Based upon behavior data collected from 28 individuals, our model predicts that the odds of correctly identifying the expressed emotion of a face reduced 16.2% when younger faces (condition 1) are artificially aged (condition 3). Replacing the younger faces with natural old-looking faces (Condition 2), however, results in an even stronger effect (odds of correct identification decreased by 50.9%). Counterintuitively, making old faces (Condition 2) look young (Condition 4) results in the largest negative effect (odds of correct identification decreased by 74.8% compared with natural young faces). Taken together, these results suggest that both age-related decline in the facial muscles’ ability to express facial emotions and age-related physical changes in the face, explain why it is difficult to recognize facial expressions from older faces; the effect of the former, however, is much stronger than that of the latter. Facial muscle exercises, therefore, might improve the capacity to convey facial emotional expressions in the elderly.

## Introduction

The ability to recognize facial emotional expression is an important social skill. Many studies have focused on characterizing the age-related decrease in ability to distinguish different facial expressions (Calder et al., 2003; Grainger et al., 2017); however, the ability to convey ones emotional state, through facial expression, has also been shown to decline with age (Borod et al., 2004; Hess et al., 2012). Not being able to signal emotions such as anger, sadness, or disgust can reduce the quality of interpersonal communication, which, not unlike hearing loss (Weinstein and Ventry, 1982), can result in social isolation. Understanding the underlying psychological and biological mechanisms behind this age-related deficit might make it easier to train social care personnel in assisting elderly, and design strategies to improve conveying emotional states in the elderly. There is thus a need to determine why it is more difficult to read the emotional expressions from older faces compared with those of younger faces.

**FIGURE 1:**
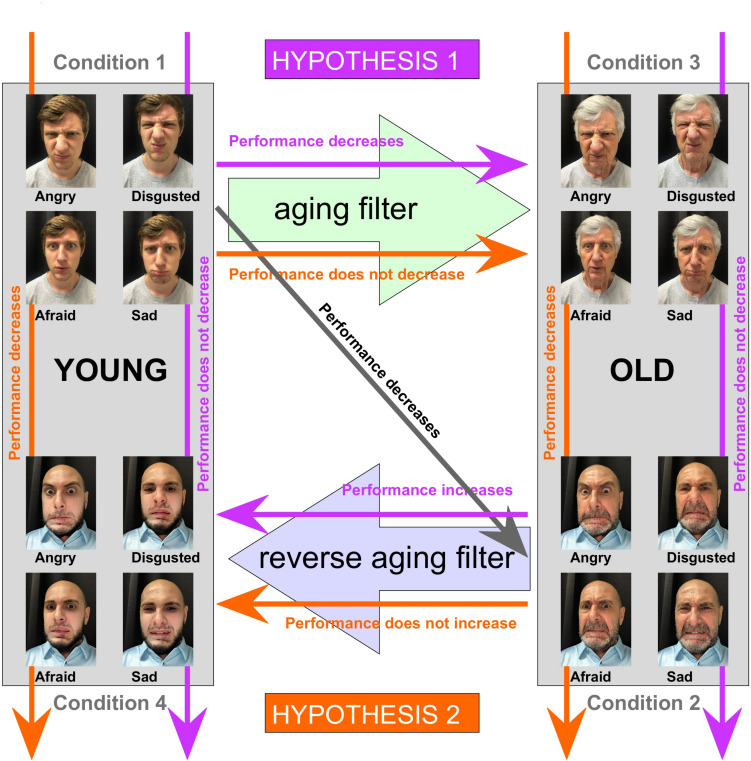
Example stimuli and schematic representation hypotheses. ***Top Left:*** an example set (not used for the actual experiment, but representative) to illustrate the stimuli employed for Condition 1, showing a young adult posing for four different negative facial expressions (sadness, disgust, fear, and anger; the neutral expression, also used for the experiment is not shown). ***Top right:*** example set to illustrate how the stimuli for condition 3, using an aging filter, were generated. ***Bottom right:*** an example set (not used for the actual experiment, but representative) to illustrate the stimuli employed for Condition 2, showing an older male posing for four different negative facial expressions (sadness, disgust, fear and anger; the neutral expression, also used for the experiment is not shown). ***Bottom left:*** example set to illustrate how the stimuli for condition 4, using a reversed aging filter, were generated. Colored arrows (purple and orange) and associated text indicate the expected effects on the participant’s ability to identify the correct facial expression in either of two cases: if physical age-related changes such as wrinkles and appearance of the skin hampers identification of the emotional expression (Hypothesis 1, purple), or if weakness of muscle, caused by aging, makes it harder to produce recognizable facial expressions (Hypothesis 2, orange).

Fölster et al. (2014) provided an extensive review of this topic and discussed the potential causes that might make it harder to correctly identify emotions expressed by an older face. The two most likely explanations are (a) the emotion expressed by weathered faces are harder to interpret because the wrinkles and folds function as distractors, masking the displayed emotion and/or (b) older individuals simply have more difficulties controlling the many facial muscles involved in emotional expression, which gradually become more inflexible with age. To discriminate between these two potential explanations, one would ideally be able to control the variables, i.e., images of young-looking faces, where the emotions are either generated by youthful or aged facial muscles, and conversely, images of old-looking faces where the expression was also generated by either youthful or aged facial muscles. Although these combinations (old face with young facial muscles and young face with old facial muscles) do not exist in the real world, we can generate visual stimuli, artificially, that mimic these specific conditions. Figure 1 shows an example of a set of stimuli where a young adult poses for different facial expressions (Condition 1). By using an aging filter, the appearance of the face is changed (Condition 3), while the key face features of a given facial expression, such as shape and position of eyes, brows, lids, nostrils, and lips are conserved; thus, an old face with the ability to express facial emotion of that of a young individual. Those key face features involved in emotional expression are also conserved when original photos of an older individual (Condition 2) are transformed using a reverse aging filter, so that the face looks a lot younger (Condition 4); in this case, the ability to express facial emotions matches that of an older person.

Transformation of the faces is achieved using a technique called generative adversarial networks (GANs), first proposed by Goodfellow et al. (2014). Unlike classical deep neural networks, great at sorting images in different categories, this revolutionary new approach allowed the creation of new images. The goal of a generative model is to study a collection of training examples and learn the probability distribution that generated them (Goodfellow et al., 2020). The technique has matured in recent years, and conditional GANs have been developed that target the generation of faces of a given type, such as face transformations that preserve identity and facial expression, or algorithms that enables editing of single attributes, such as facial hair or skin color. Applications that utilize GANs use different GAN architectures for each of the functionality they provide. To age the faces, for instance, a Contextual GAN (C-GAN) is used that generates images that fit the real face distributions, but conditioned on an individual age group. This is achieved by “feeding” countless examples (training data) to the artificial neuronal networks, “teaching” it to how the skin for a particular age group is supposed to look.

Popular publically available applications such as FaceApp, Meit5u, Oldify, Aging Booth, InkHunter, Genies, and Face Swap Life use those techniques to generate highly realistic transformations. We made use of the first to generate the different conditions for this project.

Those four conditions set up an experiment that has the potential to answer whether it is the aging face or the weakened muscles that makes it harder to identify emotional expressions in an older face. If it is the former, then participants should display superior performance identifying the emotions from younger looking faces, regardless of whether these are original faces or faces artificially transformed to look young (Hypothesis 1, Figure 1, purple arrows). On the other hand, if weakened muscles were the culprit, then we would expect that the aging or reverse aging would have little or no effect on performance. In addition, identification of emotional expression would be easy for both the original young faces and the altered version to make those faces look old, while harder for the original older faces, even if a reverse aging filter is used to make those faces look young (Hypothesis 2, Figure 1, orange arrows).

## Materials and Methods

### Subjects

Twenty-eight undergraduate students (five males and twenty-three females) received class-credit for an intro to psychology course, in return for participation in this study. Each student gave informed consent and no student participated more than once.

### Stimuli and Experimental Design

The visual stimuli used for this study were selected from FACES (Ebner et al., 2010), a database of facial expressions in younger, middle-aged, and older women and men, freely available for usage in scientific research. For Condition 1 (young), we selected the photos of 20 young individuals (10 females and 10 males), each posing for a neutral and four different negative facial expressions (angry, afraid, disgusted, and sad), thus totaling 100 different images. The FACES website which can be accessed at: https://faces.mpdl.mpg.de/imeji/, also provides picture-specific normative ratings for each image (rated by 154 individuals) which allowed us to compute the average estimate of the perceived age of all faces used for Condition 1: 29.2 ± 3.7 years. Figure 1 (see Condition 1) shows the four negative facial expressions displayed by a photographic model that is similar to those selected from the FACES database. We also selected 100 photos (10 females and 10 males; five expressions each) of older faces for Condition 2 (old), with an averaged perceived age of 67.0 ± 3.5 years. Figure 1 (see Condition 2) shows the four negative facial expressions displayed by a photographic model that is representative for the older models selected from the FACES database. To create the images for Condition 3 (young to old), we applied an aging filter (FaceApp; Wireless Lab, Skolkovo, Russia) to all the images of Condition 1, so that the faces looked older. Figure 1 (see Condition 3) illustrates the results yielded by applying the aging filter to the examples used to illustrate Condition 1. A reversed aging filter was used to make the faces used for Condition 2, look younger (Condition 4, old to young). Figure 1 (see Condition 4) illustrates the results yielded by applying the reverse aging filter to the examples used to illustrate Condition 2.

For each session, the 400 unique images (four conditions × 100 images) were displayed, sequentially, in a random order. The order of presentation was thus different for each participant. Each image was presented at the center of the screen (17° × 23° of visual angle). Subjects were instructed to identify the facial expression of the faces that were viewed, using a button box with five buttons labeled angry, afraid, disgusted, sad, or neutral. Images disappeared from the screen upon evaluation (button press), followed by a 1-s inter-trial interval, during which a cross was displayed in the center of the screen, before the next image appeared. All participants were encouraged to respond as quickly and as accurately as possible; images not evaluated within 4 s disappeared from the screen.

To make the participants familiar with the task, they first performed practice trials featuring 20 images not used for the actual experiment.

All stimuli were presented on a Dell 19-inch monitor, placed 50 cm from the participants’ heads. The code for the experimental paradigm was generated by using experiment builder (SR Research). This project was approved by the institution’s IRB. The data that support the findings of this study are openly available in figshare at: https://doi.org/10.6084/m9.figshare.14043797.v1.

### Analyses

Analyses were conducted using IBM SPSS Statistics, Version 27 (IBM Corp., Armonk, NY, United States).

A generalized linear mixed model using binary logistic regression was employed to predict participant accuracy identifying the displayed facial expression (Level 1 binary outcome variable of correct or incorrect answer). Model 1 is an unconditional model that included only answer accuracy as the outcome and participants as the Level 2 subjects because of the repeated trials they performed in the study, but no predictor variables. The variation in intercepts demonstrates the variation in proportion of subject answer accuracy. Model 2 added Level 1 fixed predictors which included type of face (young, young to old, old, and old to young; reference = young), as our main hypotheses are focused on this variable. Model 3 added on additional Level 1 fixed predictor of type of expression (angry, afraid, disgust, sad, and neutral; reference = neutral) and also allowed the slopes of photographic model to randomly vary between participants, our Level 2 subject variable. Using photographic models as a random fact seemed prudent, as we were interested in determining how much of the overall variation in responses was attributed to this factor.

## Results

### Generalized Linear Mixed Model Using Binary Logistic Regression

Model 1 was the unconditional model that did not include any predictor variables. The model was able to classify cases correctly 76.9% of the time, and it determined that the odds of a participant selecting the correct facial expressed was 3.4 times greater than the odds of the participant selecting an incorrect answer (see Table 1). The variance estimate for random participant intercepts was 0.117, which demonstrated that there was a significant difference (*z* = 3.276, *p* = 0.001) between the log odds proportion of correct answers given by each participant. This also indicates that there may be additional predictors that could be added to the model that could help explain the random variation.

**TABLE 1:** Results of logistic regression for accurate identifications of facial expressions.

						**95% CI for odds ratio**	
**Variable**	**Coefficient**	**SD**	***t***	***p***	**Odds ratio**	**Lower**	**Upper**
**Model 1**							
Intercept	1.225	0.068	18.138	<0.001	3.403	2.981	3.885
**Model 2**							
Intercept	1.783	0.094	19.065	<0.001	5.949	4.952	7.145
*Face Type*							
Young to old	–0.161	0.054	–2.982	0.003	0.851	0.766	0.946
Old to young	–1.209	0.093	–13.072	<0.001	0.299	0.249	0.358
Old	–0.642	0.057	–11.251	<0.001	0.526	0.471	0.589
**Model 3**							
Intercept	4.063	0.238	17.048	<0.001	58.172	36.459	92.817
*Face type*							
Young to old	–0.177	0.058	–3.023	0.003	0.838	0.747	0.940
Old to young	–1.377	0.108	–12.742	<0.001	0.252	0.204	0.312
Old	–0.712	0.063	–11.381	<0.001	0.491	0.434	0.555
*Expression*							
Anger	–2.748	0.240	–11.435	<0.001	0.064	0.040	0.103
Afraid	–1.366	0.213	–6.399	<0.001	0.255	0.168	0.388
Disgust	–2.808	0.227	–12.375	<0.001	0.060	0.039	0.094
Sad	–2.681	0.268	–10.015	<0.001	0.069	0.041	0.116

Model 2 included the Level 1 fixed predictors variables of face type (young, young to old, old, and old to young). The regression was able to correctly classify 77.1% of cases. The inclusion of face as a fixed factor was a significant predictor in the model (see Table 2) and compared to the young face reference group, all other types of faces were significantly less likely to have their emotional expressions identified correctly (see Table 1). The variance estimate for random participant intercepts was 0.129, which demonstrated that there was a significant difference (*z* = 3.293, *p* = 0.001; see Table 3) between the log odds proportion of correct answers given by each participant and still may indicate that additional predictors added into the model could explain more of the random variation.

**TABLE 2:** Fixed effects in logistic regression model.

**Source**	***F***	**df 1**	**df 2**	***p***
**Model 2**				
Corrected model	66.067	3	10,995	<0.001
Face	66.067	3	10,995	<0.001
**Model 3**				
Corrected model	38.609	7	10,991	<0.001
Face	64.511	3	10,991	<0.001
Emotional expression	47.222	4	10,991	<0.001

**TABLE 3:** Random effects in logistic regression model.

**Variance**	**Estimate**	**SD**	***z***	***p***	**Lower**	**Upper**
**Model 1**						
Intercept (subjects)	0.117	0.036	3.276	0.001	0.065	0.214
**Model 2**						
Intercept (subjects)	0.129	0.039	3.293	<0.001	0.071	0.234
**Model 3**						
Intercept (subjects)	0.164	0.050	3.257	0.001	0.090	0.300
Photographic models (subjects)	0.151	0.036	4.220	<0.001	0.095	0.240

Model 3 included emotional expression as an additional significant fixed predictor (see Table 2) and photographic model as a random effect where slopes were allowed to vary between participants. The regression was now able to correctly classify 80.0% of cases, and all face types and all emotions were significantly less likely to identify the expressed emotion correctly when compared to their respective reference groups (young faces for face type and neutral emotion for expression). The random participant intercepts changed to 0.164 in this analysis version, which still demonstrated a significant difference between the log odds proportion of correct answers given by each participant (*z* = 3.257, *p* = 0.001). There was significant variation in the slopes of photographic models with an intercept of 0.151 (*z* = 4.220, *p* < 0.001). Although these values were significant, the intraclass correlation coefficients (ICC) were 0.047 and 0.043, respectively, which are below the 0.05 threshold that is conventionally regarded as indicating more evidence of clustering (Heck et al., 2014).

## Discussion

### Hypothesis 1 Versus Hypothesis 2

As expected based upon previous studies (Hess et al., 2012; Freudenberg et al., 2015), our research participants had more trouble identifying the expressions displayed by older faces compared with younger faces. According to our analytical models (see Table 1), the odds of selecting the correct emotion significantly diminishes when the face to be assessed is old (condition 1) instead of young (condition 2). With this established difficulty in mind, we designed an experiment to discriminate between two potential explanations, formulated two hypotheses, and described the expected behavior that would match each hypothesis (Figure 1). Although artificially aged faces also decreased the odds of selecting the correct answer (16.2% when compared to young faces according to model 3), those effects were a lot smaller than what was observed when the artificially aged faces were replaced by “natural” old faces (odds of correctly identifying the emotion was reduced by 50.9% when compared to young faces according to model 3). Hypothesis 1 predicts that rejuvenating older faces would reverse those reduced odds, but the opposite was observed: making the face artificially younger reduced the odds of correct identification by 74.8% when compared to a naturally young face, according to model 3. Taken together, those results suggest that both age-related changes in the face, such as wrinkles, as well as the difficulties older individuals experience expressing emotions, explain why it is harder to read the emotion from older faces. Interestingly, the results suggest that the effect of the latter is much stronger than that of the former. The parsimonious explanation for these findings is that with age, the facial muscles undergo atrophy, limiting an individual’s ability to generate emotional expressions, which in turn makes it harder for an observer to read the intended emotional expression. In contrast, other studies (Hess et al., 2012; Freudenberg et al., 2015) identified the wrinkles and folds as being responsible for the reduction in emotion perception. Neither of those studies, however, were designed to identify the exact cause of this age effect.

### Facial Emotional Expression

Our study was designed with the aim of testing the hypotheses formulated in the introduction, thus based upon face type rather than directly testing the effect of the displayed emotion. Our analyses, however, revel that each expressed emotion significantly reduces the odds of correct identification when compared with the neutral expression. It is sometimes challenging to compare the accuracy of identifying facial expressions of emotions between studies, because different studies use different forced-choice options; in addition, the particular face database used has also been shown to be an important variable (Dores et al., 2020). Yet, we identified several studies (Calvo and Lundqvist, 2008; Dores et al., 2020), where negative emotions were found to be harder to identify compared with neutral faces, thus consistent with our results. Hess et al. (2012) reported main effects for faces (old vs young), emotions (happy, angry, and sad), and emotion by facial age interaction. A study to further explore the interactions between facial age and expressed emotion requires a design that also includes positive emotional expressions.

### Alternative Explanation

Some studies found that negative affect decreases with age, as older adults tend to report experiencing more positive emotions (Scheibe and Carstensen, 2010). Whether experiencing negative emotions as less intense and/or less arousing also results in a reduced ability to produce negative emotional facial expressions, and hence offer an alternative explanation for our results, is not known. Another intriguing possibility, consistent with our results, is that the causality is reversed; namely that emotions in older individuals are experienced as less intense because their facial expression directly affects their emotional experience, i.e., the facial feedback hypothesis (McIntosh, 1996).

Further investigation is thus required to establish the exact causality.

### Limitations

Our participants were college-aged male and females; we can therefore not exclude an own-age bias (Fölster et al., 2014). However, since artificial aging of the faces had little effect on emotional recognition, while reversed aging of older faces decreased, rather than improved, emotional recognition, there is unlikely a significant own-age bias. A meta-analytic study, however, has found that participants display a better recognition memory for faces that match their own age group than those matching a different age group (Rhodes and Anastasi, 2012). Another well-established effect in face perception is that of the own-race bias. Unfortunately, our face dataset only contains Caucasian faces, which we recognize as a regrettable limitation. The challenge for our study was to find a face database with photographic models from different age groups, posing for different emotional expressions. Since 2018, a racially diverse affective expression face stimulus database has been freely available for researchers (Conley et al., 2018); however, since the photographic models who posed for this particular database all belong to roughly the same age-group, it was not suitable for our study. Repeating the study with racial diverse photographic models and research participants that are more representative of the public, will inform whether the results of our study can be generalized.

Our study makes use of images that we modified using advanced GAN. The results look very convincing and realistic, and although it is a difficult task to discriminate those deep fakes from real images, it is not impossible (Guarnera et al., 2020). Whether using GANs [see also our previous study (Brunet and Sharp, 2020)] to create chimeric combinations (young face + aging facial muscles; old face + youthful facial muscles) is a legitimate technique to study facial expression is of course always a valid concern. Related techniques to control for variables such as the creation of morphs (Gutiérrez-Maldonado et al., 2014) and computer-generated virtual faces (Souto et al., 2013; Cortes et al., 2019) have been used chiefly in face perception studies.

## Conclusion

Using real images of facial expressions, as well as GANs to make the original images look younger or older, we conducted a study that demonstrates that observers struggle to read the emotional expression from older faces more than they do from younger faces. Whereas other studies (Hess et al., 2012; Freudenberg et al., 2015) ascribe this deficit to the wrinkles and folds that are characteristic of the age-related weathering of the face, our study suggests that it is mainly the facial muscles’ ability to generate emotions–which declines with age–that complicates the interpretation of facial expressions.

These findings suggest that the elderly might benefit from facial muscle exercises. A recent study (Hwang et al., 2018) reported that facial muscle exercises using a Pao device significantly increased facial muscle thickness and cross-sectional area. Whether strategies that focus on maintaining healthy facial muscles improve the ability to generate distinct emotional expressions in the elderly, thus potentially increasing emotional interpretability, is an exciting area for further investigation.

## Data Availability Statement

The raw data supporting the conclusions of this article will be made available by the authors, without undue reservation.

## Ethics Statement

The studies involving human participants were reviewed and approved by The Millsaps College Human Subject Review Board (HSRB Project Number #2019-28). The patients/participants provided their written informed consent to participate in this study.

## Author Contributions

SG conducted analyses, contributed with the interpretation of the data and the results, and with the writing of the revised manuscript. NB designed the experimental paradigm, conducted preliminary analyses, and wrote the manuscript. AJ generated the stimuli, recruited participants, and collected data. CT recruited participants and collected data. All authors contributed to the article and approved the submitted version.

## Conflict of Interest

The authors declare that the research was conducted in the absence of any commercial or financial relationships that could be construed as a potential conflict of interest.
